# Isolation and characterization of the new *Streptomyces* phages Kamino, Geonosis, Abafar, and Scarif infecting a broad range of host species

**DOI:** 10.1128/spectrum.00663-24

**Published:** 2024-09-25

**Authors:** Bente Rackow, Clara Rolland, Isabelle Mohnen, Johannes Wittmann, Mathias Müsken, Jörg Overmann, Julia Frunzke

**Affiliations:** 1 Institute of Bio- and Geosciences, Forschungszentrum Jülich, Jülich, Germany; 2 Leibniz Institute DSMZ—German Collection of Microorganisms and Cell Cultures, Braunschweig, Germany; 3 Central Facility for Microscopy, Helmholtz Centre for Infection Research, Braunschweig, Germany; University at Albany, Albany, New York, USA

**Keywords:** phage-host interaction, *Streptomyces*, phage defense

## Abstract

*Streptomyces*, a multifaceted genus of soil-dwelling bacteria belonging to the phylum *Actinomycetota*, features intricate phage-host interactions shaped by its complex life cycle and the synthesis of a diverse array of specialized metabolites. Here, we describe the isolation and characterization of four novel *Streptomyces* phages infecting a variety of different host species. While phage Kamino, isolated on *Streptomyces kasugaensis*, is predicted to be temperate and encodes a serine integrase in its genome, phages Geonosis (isolated on *Streptomyces griseus*) and Abafar and Scarif, isolated on *Streptomyces albidoflavus*, are virulent phages. Phages Kamino and Geonosis were shown to amplify well in liquid culture leading to a pronounced culture collapse already at low titers. Determination of the host range by testing >40 different *Streptomyces* species identified phages Kamino, Abafar, and Scarif as broad host-range phages. Overall, the phages described in this study expand the publicly available portfolio of phages infecting *Streptomyces* and will be instrumental in advancing the mechanistic understanding of the intricate antiviral strategies employed by these multicellular bacteria.

**IMPORTANCE:**

The actinobacterial genus *Streptomyces* is characterized by multicellular, filamentous growth and the synthesis of a diverse range of bioactive molecules. These characteristics also play a role in shaping their interactions with the most abundant predator in the environment, bacteriophages—viruses infecting bacteria. In this study, we characterize four new phages infecting *Streptomyces*. Out of those, three phages feature a broad host range infecting up to 15 different species. The isolated phages were characterized with respect to plaque and virion morphology, host range, and amplification in liquid culture. In summary, the phages reported in this study contribute to the broader collection of publicly available phages infecting *Streptomyces*, playing a crucial role in advancing our mechanistic understanding of phage-host interactions of these multicellular bacteria.

## INTRODUCTION

*Streptomyces*, a genus of soil-dwelling multicellular Gram-positive bacteria belonging to the phylum *Actinomycetota*, stands out for its extensive array of biosynthetic gene clusters. These clusters encode a wide range of specialized metabolites exhibiting diverse biological activities, such as antibacterial, antifungal, anticancer, and even antiviral properties. Today, up to two-thirds of all nature-based antibiotics used in clinics are produced by *Streptomyces* spp. (1–3). Furthermore, the intricate life cycle of *Streptomyces* positions it as a model for the study of multicellular development in bacteria (4). Microbial interaction was shown to be a prominent trigger of secondary metabolite production and cellular development. Co-cultivations of microorganisms therefore represent a promising approach for the discovery of novel antibiotics otherwise not produced under laboratory conditions (5). In recent years, also the interaction of *Streptomyces* with their most abundant predator in the environment—bacteriophages (or phages for short)—increasingly gained attention (6, 7).

Phage research in previous decades focused on phages for tool development employing phages like phiC31 or R4 (8–10). Genetic tools such as restriction enzymes and integrative plasmids have been constructed and are still valuable tools used in *Streptomyces* genetics (11, 12). In recent years, however, the focus of actinobacteriophage research has shifted from tool design to phage-host interaction, in particular to the elucidation of novel antiviral defense mechanisms (13). Remarkably, it was shown that secondary metabolites produced by *Streptomyces*, belonging to the classes of anthracyclines and aminoglycosides, inhibit infection by a broad range of phages (14, 15). Furthermore, cellular development was shown to play a key role in the emergence of transient resistance to phage infection. This was shown for the development of transiently resistant mycelium at the infection interface on plates (16) as well as for the formation of S-forms lacking the cell wall, which occurred during infections under osmoprotective conditions (17).

For the discovery and mechanistic understanding of novel defense strategies, a diverse set of phages is needed, incentivizing the isolation and characterization of new phages. Using different *Streptomyces* species as isolation host, several new phages have been isolated and described in recent years (18–21). These studies show that the common notion of a narrow host range and specific infection of phages does not necessarily apply to *Streptomyces* phages, as several actinophages have been described to have a broader host range and to productively infect several species of the genus (20, 22). However, in most studies, only a small number of different host species has been tested, limitating the information regarding host range.

In this study, we isolated and characterized four new phages infecting *Streptomyces*. Testing a collection of over 40 distinct *Streptomyces* strains, we observed that the phages Kamino, Abafar, and Scarif exhibited the ability to infect a wide array of *Streptomyces* species. This characteristic emphasizes their suitability for comparative studies on phage defense mechanisms across various host species. All four phages isolated in this study were sequenced and characterized according to their infection dynamics and morphology.

## MATERIALS AND METHODS

### Bacterial strains and growth conditions

*Streptomyces albidoflavus* M145, *Streptomyces griseus,* and *Streptomyces kasugaensis* were used as main host strains in this study. Bacterial cultures were started from a spore stock, stored at –20°C in 20% glycerol. Spores were inoculated into fresh liquid Glucose Yeast Malt extract (GYM) medium for *S. griseus* and *S. kasugaensis* or into Yeast extract Malt extract (YEME) medium for *S. albidoflavus* M145. In general, cultivation was carried out at 30°C. Double agar overlays were performed using GYM agar for all species, were 4 mL 0.4% agar and 20 mL 1.5% agar was used for the top and bottom layer, respectively.

### Phage isolation and propagation

Phages described in this study were isolated from soil samples taken in the Eifel region (Aachen, Germany) and Braunschweig, respectively. The soil samples were incubated with sodium chloride/magnesium-sulphate (SM) buffer (10 mM Tris-HCl pH 7.3, 100 mM NaCl, 10 mM MgSO_4_, 2 mM CaCl_2_) over night at 4°C on a shaking plate, to resolve phages from soil particles (18). The samples were then centrifuged for 30 min at 5,000 × *g* to separate the supernatant from soil particles. The supernatant was subsequently filtered through a 0.22 µm pore size membrane filter, to remove remaining bacteria. The lysate was furthermore enriched on the host bacteria described above, to enrich potential phages overnight. After overnight incubation, serial dilutions were spotted on a bacterial lawn propagated by mixing overnight cultures of the host strains with 4 mL top agar to a final OD_450_ = 0.4. Plaques were visualized after incubation over night at 30°C.

Pure phages were obtained by restreaking single plaques twice on a double agar overlay. Amplification of phages for high titer stocks was performed by mixing 100 µL phage sample with the top agar of a double agar overlay to obtain near confluent lysis with remaining “spider web” of the bacterial lawn. After overnight incubation at 30°C, the phages were resolved by adding 5 mL SM buffer to the plates and shaking the agar plates for 1–2 h at room temperature at 40 rpm. The SM buffer was then collected from the plates, centrifuged, and subsequently filtered through a 0.22 µm membrane filter to separate remaining bacteria from the phage lysate. The high titer lysate was mixed with 10% (vol/vol) sterile glycerol and stored at either −80°C for long-term storage or 4°C for further experiments. The phage titer of the lysate was determined by spotting 2 µL of a serial dilution up to 10^−8^ onto a double agar overlay with the top agar containing the host bacterium to an OD_450_ = 0.4. After overnight incubation at 30°C, the highest dilution with visible single plaques was counted and the titer in plaque forming units (PFU) per milliliter was calculated. The isolated phages are available in the DSMZ strain collection, Abafar (DSM 115547), Geonosis (DSM 114972), Kamino (DSM 114973), and Scarif (115542).

### Electron microscopy

For transmission electron microscopy (TEM), 5 µL of pure high titer phage lysate was dropped on a glow discharged (15mA, 30 s) carbon-coated copper grid (CF300-CU, carbon film 300 mesh copper). The phage containing grid was stained with 2% (wt/vol) uranyl acetate for 5 min and washed twice in ddH_2_O. Dried samples of Kamino and Geonosis were analyzed with a TEM Talos L120C (Thermo Scientific, Dreieich, Germany) at an acceleration of 120 kV. Abafar and Scarif samples were examined in a Zeiss EM 910 or Zeiss Libra120 Plus transmission electron microscope (Carl Zeiss, Oberkochen, Germany) at an acceleration voltage of 80 kV/120 kV at calibrated magnifications using 300 mesh copper grids and a mica-floated carbon film enabling attachment of phages.

### Infection dynamics

Infection experiments in liquid cultures were performed as described in Hardy et al. (18). Cultivation was performed in the BioLector micro cultivation system of m2plabs (Aachen, Germany) as biological triplicates in 48-well flower plates (m2plabs) at 30°C with a shaking frequency of 1,200 rpm. Backscattered light intensity was measured every 15 min (filter module: e_xcitation_/e_mission_, 620 nm/620 nm; gain 25), and supernatant samples were taken every 2 h to assess the infection dynamics and amplification rate. Infection took place in 1 mL YEME or GYM medium with *S. albidoflavus* M145*, S. griseus,* and *S. kasugaensis* with an initial OD_450_ of 0.15. Phages were directly added to the cells with initial titers from 10^2^ to 10^7^ PFU/mL. The sampled supernatant was centrifuged to separate phages from cell remnants and subsequently diluted in a serial dilution of 10^−1^ to 10^−8^. The dilutions were spotted on a GYM double agar overlay containing the respective host bacteria in the top agar layer with an OD_450_ = 0.4 to determine the phage titer over the course of the infection.

### Plaque development

The plaque morphology of the phages Abafar, Geonosis, Kamino, and Scarif were observed on double agar overlay plates with a titer of 10^2^ to 10^3^ PFU/mL of each phage and an OD_450_ = 0.4 of the respective host bacterium in the top agar. The plaque assay plates were incubated at 30°C for 72 h in total, and images of single plaques were taken using a Nikon SMZ18 stereomicroscope with NIS-Elements AR 5.3 software after 24 h, 48 h, and 72 h. The area of the plaques were determined by using the auto detect ROI (region of interest) tool of the NIS-elements software and the area of the ROI was calculated automatically.

### Phage-host range assay

Host-range determination of the four phages was performed with classical spot assays as described in the section “Phage isolation and propagation.” Different *Streptomyces* species (see Table S1 for productive infections and Table S2 for all tested strains) were used as bacterial lawn to test the infectivity of the respective phages. In this study, we discriminated between the simple clearance of the bacterial lawn by the phages and truly productive infections, where single plaques of the phages could be observed on the bacterial lawn. For all productive infections, the efficiency of plating (EOP) was calculated by dividing the counted plaques on the tested bacterial host by the counted plaques on the “original” host, which was used for isolation.

### DNA isolation, genome assembly, and annotation

Genomic DNA of the phages was isolated from 1 mL phage lysate with a high titer using the Norgen Biotek Corp. Phage DNA isolation Kit (Thorhold, Canada). Isolation was carried out as described in the manual provided by the manufacturer, including all optional steps. To increase the concentration, a two-step elution of the DNA in each 25 µL elution buffer was performed. DNA concentration was measured in a NanoPhotometer (P330, IMPLEN, Germany). Whole genome sequencing using the Illumina NovaSeq platform with a read length of 2 × 150 bp was performed by GENEWIZ Germany. The NEBNext Ultra II DNA Library Prep Kit was used to sequence the whole genome of phages on an Illumina MiSeq platform with paired reads of 15–150 base pairs in length. Initially, quality control checks for each pair of raw sequencing reads were performed using FASTQC v.0.11.9 (http://www.bioinformatics.babraham.ac.uk/projects/fastqc/). The adapter and low-quality reads were cleaned and trimmed from the sequencing data with the help of the fastp v.0.23.2 program (23). Next, the whole genome *de novo* assembly with the trimmed high-quality reads was performed with the help of the Shovill pipeline v.1.1.0 (https://github.com/tseemann/shovill) using the SPAdes genome assembler v.3.15.5 (24). Lastly, Pilon version 1.24 (25) was used to improve and curate the assembled genomes. Phage genomic terminal ends were identified using the PhageTerm (26) online galaxy platform.

All phage genomes were annotated with Prokka 1.8 using different databases (Markov model profile databases, including Pfam and TIGRFAMs). A search was performed using hmmscan from the HMMER 3.1 package (27). Sequences were also compared to the PHROG database (28). Taxonomic classification was determined on the identity level to close related phages using NCBI BLASTn (NCBI, 2023) search and ordered according to the new virus taxonomy release (International Committee on Taxonomy of Viruses, 2023). All phage genomes were deposited at the NCBI database with the GenBank accession numbers Abafar (PP750865), Geonosis (PP750866), Kamino (PP750867), and Scarif (PP750868).

## RESULTS

### Bacteriophage isolation and morphology

**Fig 1:**
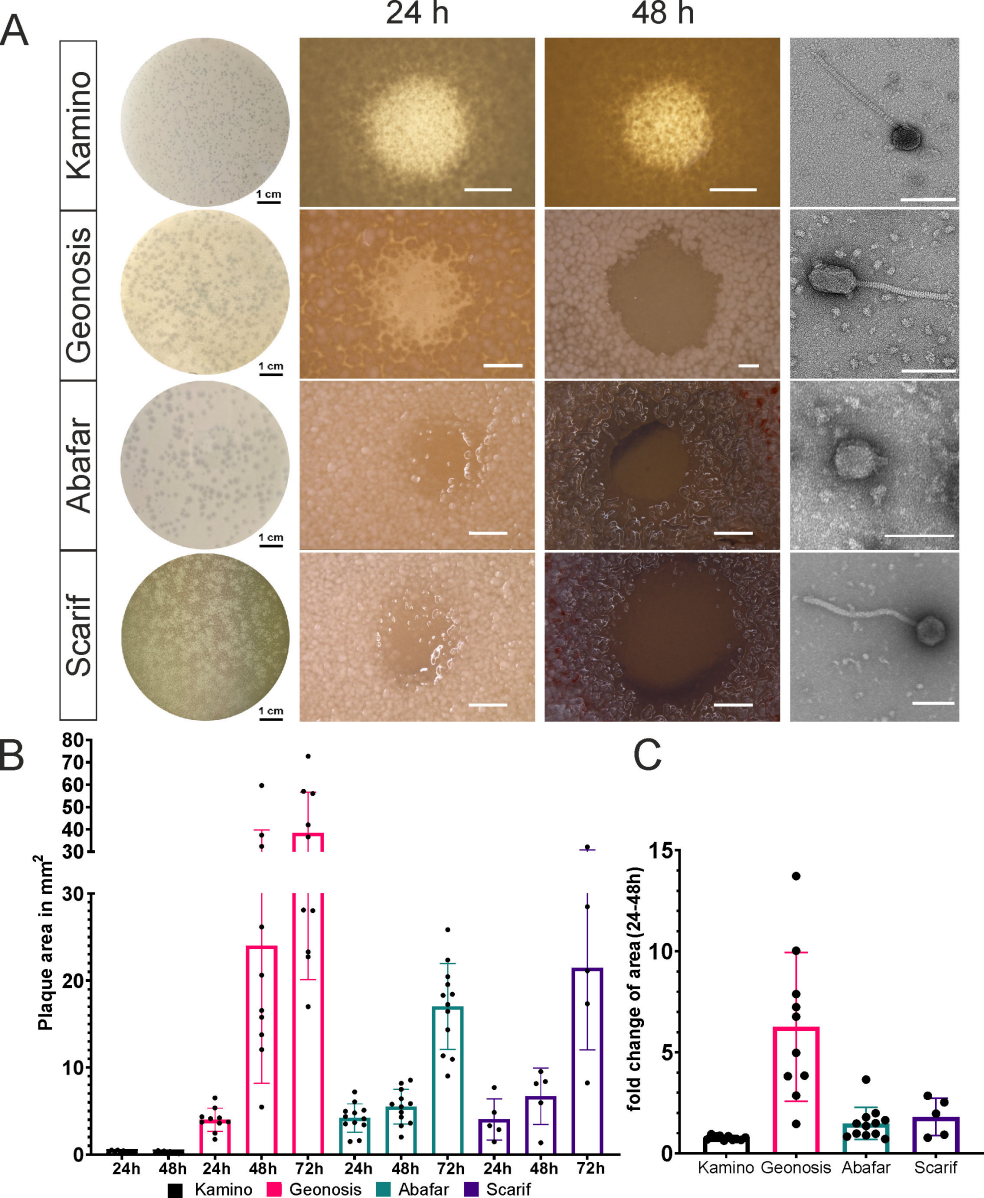
Morphological comparison of plaques and virions. (**A**) Plaque and TEM images of the tested phages with an overview image of a plate with plaques 24 h post infection, a plaque close-up image 24 h and 48 h post infection, and a TEM image of the virion from left to right, respectively, for the phages Kamino, Geonosis, Abafar, and Scarif from top to bottom. The scale bar for the overview images is 1 cm in length, for plaque close-ups, the scale bar is 1,000 µm, and for TEM images, 100 nm. (**B**) Comparison of the development of plaque area over time of 24–72 h post infection with plaque area shown in square millimeters for the phages Kamino (black, *n* = 10) Geonosis (pink, *n* = 10), Abafar (turquoise, *n* = 12), and Scarif (purple, *n* = 5). (**C**) Fold change of plaque area between 24 h and 48 h post infection for all four phages in the same color as graph B.

Four novel phages infecting different *Streptomyces* species were isolated from soil. Phage Kamino was isolated on *S. kasugaensi*s as host, Geonosis on *S. griseus*, as well as Abafar and Scarif on *S. albidoflavus* as isolation host. Phage Kamino forms small, turbid plaques (Fig. 1A) with a plaque area of approximately 0.3 mm^2^; the plaque size is relatively constant over 48 h of incubation (Fig. 1B). Phage Geonosis forms round and clear plaques with sharp edges (Fig. 1A) and a plaque area of approximately 4 mm^2^ after 24 h of incubation, which increases up to 38 mm^2^ on average after 72 h of incubation (Fig. 1B). The *S. albidoflavus* infecting phages Abafar and Scarif show an overall similar plaque size as Geonosis with average plaque areas of 4.2 and 4.0 mm^2^, respectively, after 24 h incubation. Abafar and Scarif also show an increase in plaque area over the course of 72 h up to an average plaque area of 17 and 21 mm^2^ (Fig. 1B). Additionally, around the plaques of Abafar and Scarif, enhanced production of actinorhodin was observed by the formation of colored halos around the plaques (Fig. S1), which has been described in previous studies reporting *S. albidoflavus/Streptomyces coelicolor* phages (18, 19) (Fig. 1A).

TEM imaging of the phage particles revealed that phages Kamino, Geonosis, and Scarif belong to the morphotype siphovirus with Kamino and Scarif showing an icosahedral capsid and Geonosis an elongated head (B3 morphotype), all three display a long, non-contractile tail, whereas phage Abafar features a podoviral morphotype, showing an icosahedral capsid with a very short tail (Fig. 1A, right column). Details on capsid and tail length are provided in Table 1.

**TABLE 1:** Morphological comparison of virions

Phage	Tail length (nm)	Capsid length (nm)	Capsid diameter (nm)
Kamino (*n* = 13)	223.4 (+/– 7.1)	63.5 (+/– 2)	61.5 (+/– 1.4)
Geonosis (*n* = 15)	176.5 (+/– 4.4)	88.6 (+/– 4.8)	53.8 (+/– 3.0)
Abafar (*n* = 30)	16 (+/– 2)	60 (+/– 3)	61 (+/– 2)
Scarif (*n* = 30)	254 (+/– 10)	69 (+/– 4)	65 (+/– 3)

### Infection curves of bacteriophages

**Fig 2:**
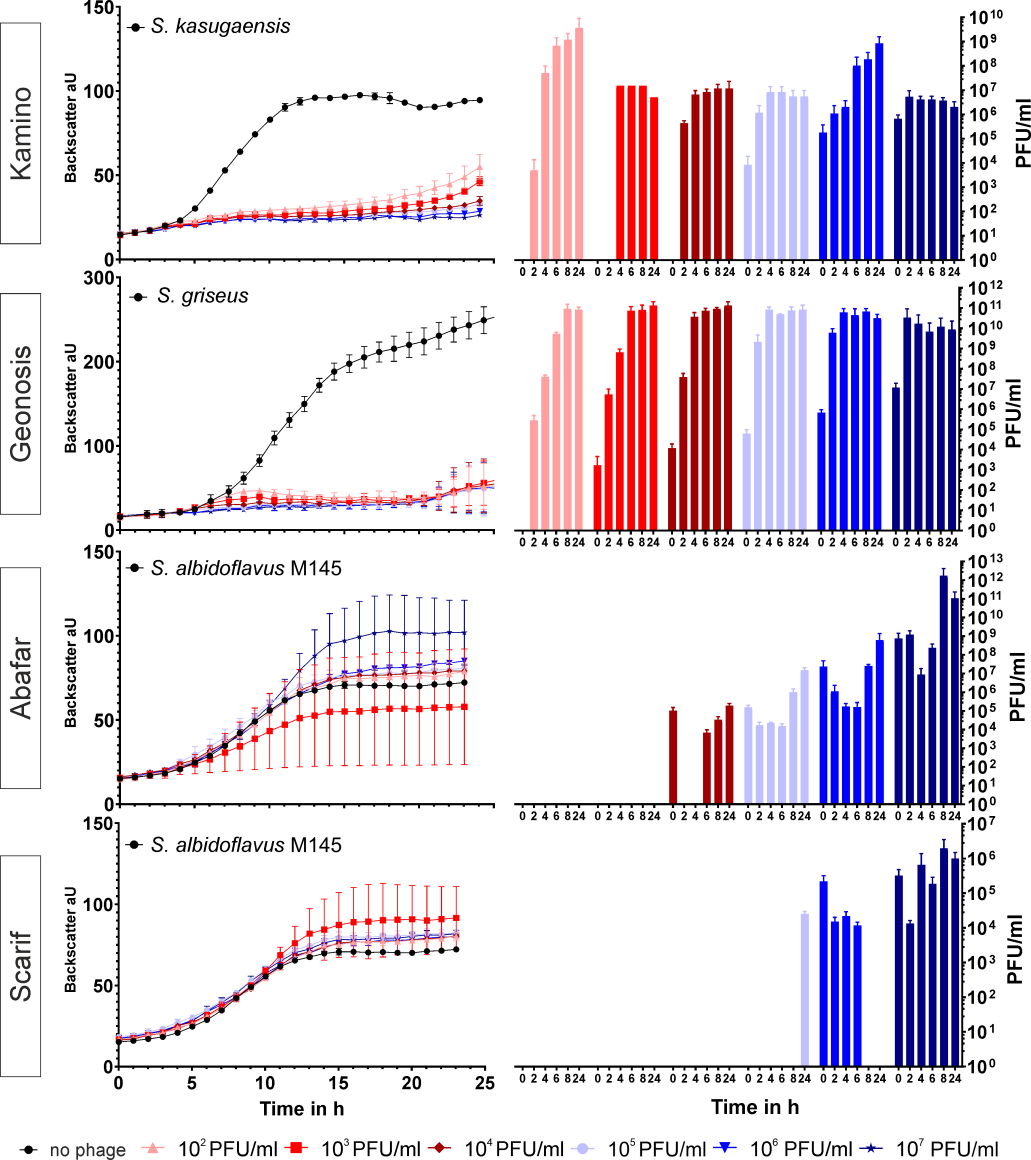
Infection curves of the four phages on their isolation host *S. kasugaensis* infected by Kamino, *S. griseus* infected by Geonosis, and *S. albidoflavus* M145 infected by Abafar and Scarif. *S. kasugaensis* and *S. griseus* were inoculated to GYM medium, and *S. albidoflavus* was grown in YEME medium. All strains were cultivated in microtiter plates and infected with increasing titers of the respective phages. In the left panel, the backscatter is plotted against time to visualize growth of the bacterial culture. In the right panel, phage titers at different time points throughout the infection (2/4/6/8 and 24 h) are shown. The colors of the growth curves and the bar plots for the phage titer correlate to the same initial infection titer between 10^2^ and 10^7^ PFU/mL, the black curve indicates growth of the host bacterium in the absence of phages. *n* = 3 independent biological replicates. Error bars indicates the standard deviation.

In order to assess phage infection dynamics, phage infections in liquid cultures on the original host strain were performed. As it is not suitable to perform one-step growth curves with *Streptomyces* spp. due to the multicellular development, the host strains were cultivated in microtiter plates in the presence of different phage titers. Cell growth was monitored by backscatter measurements in 15-min intervals over the course of 24 h and phage propagation was determined by taking samples from the culture supernatants every 2 h to determine the titer of infectious phage particles at the respective time (Fig. 2). Infections with phage Kamino and phage Geonosis lead to a complete culture collapse of their respective hosts, *S. kasugaensis* and *S. griseus,* with a starting titer as low as 10^2^ PFU/mL. The titer of Kamino and Geonosis increased steadily and reached final titers of 10^6^ to 10^9^ PFU/mL for Kamino and 10^10^ to 10^11^ PFU/mL for Geonosis. On high titers (10^7^), however, little to no amplification was observed for phage Kamino. In contrast, phages Abafar and Scarif showed little to no growth defect in their host strain. Amplification of Abafar and Scarif could only be observed in spot assays, determining the phage titer over time. In case of phage Abafar, amplification in liquid was first observed for a starting titer of 10^4^ PFU/mL and for phage Scarif at a starting titer of 10^5^ PFU/mL, which indicates a probably lower burst size and decreased infectivity compared to Geonosis and Kamino. While the final titer determined for phages Kamino and Geonosis appeared independent from the starting titer, final titers drastically increased for Abafar and Scarif, when infection was initiated with higher starting titers. Under the specified conditions, Abafar achieved final titers of 10^6^ to 10^12^ PFU/mL 24 h post infection while Scarif achieved titers between 10^4^ and 10^6^ PFU/mL. Altogether, all four phages are able to propagate in liquid cultures to different extents. Abafar and Scarif, however, reach higher titers when the lysate is prepared on plates. For phage Abafar and Scarif, it can also be observed that the phage titer in the spot assay does not necessarily correlate with the added phages. This phenomenon might be explained by the complex growth of the host bacterium *S. albidoflavus.* We hypothesize that the mycelial structures of the host might trap free phages without being infected and thereby lowering the phage titer in the supernatant.

### Host range of phage isolates

**Fig 3:**
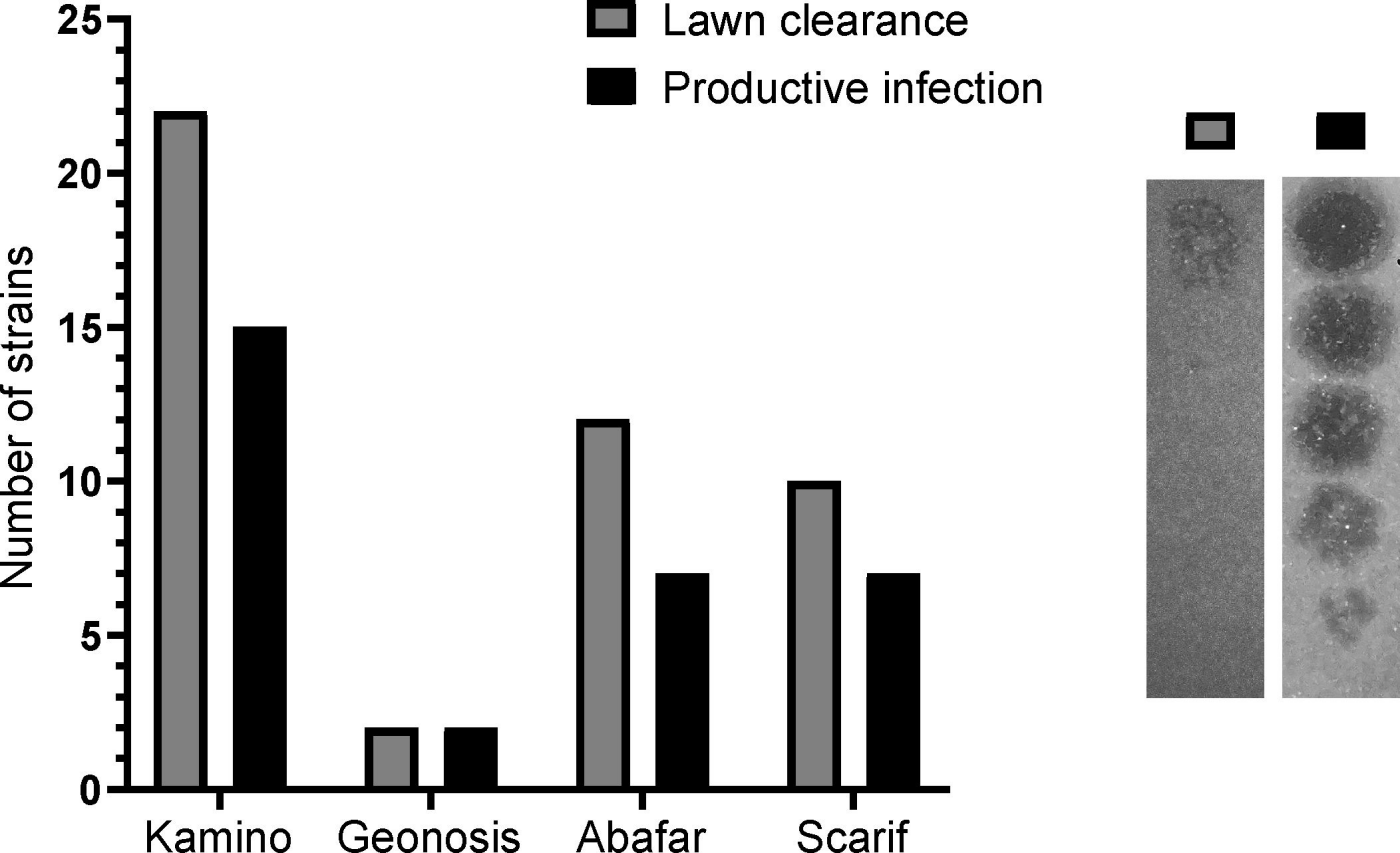
Distribution of productive infection (black) versus lawn clearance (gray). On the right are exemplary images of lawn clearance and productive infection of phage Scarif on *S. albidoflavus*.

One physiological trait that is important to consider when using or studying phages is the range of bacterial hosts they are able to infect. In this study, we determined the host ranges of the novel phage isolates by spotting serial dilutions of the phages on lawns of >40 different *Streptomyces* species (Table S2). A distinction must be made between the simple lysis of bacteria and the ability to cause a productive infection because only the latter leads to the formation of individual plaques (Fig. 3). From the four phages described in this study, phage Kamino has the broadest host range with lawn clearance on 22 *Streptomyces* spp. and productive infections on 15 different species among the 45 species tested (Table S2; Fig. 3). The EOP refers to the ratio of plaques formed on the host used for isolation compared to another host species. While phage Kamino is able to infect a wide variety of host strains, the EOP ranges from 0.002% on *Streptomyces olivaceus* up to 5,000% on *Streptomyces afghaniensis* (Table 2). The phages Abafar and Scarif have the same range of productive infections with seven different species but differ in their lawn clearance, where Abafar shows clearance on 12 species and Scarif only on 10 (Fig. 3). Phage Abafar displays an EOP lower than 100% on the alternative hosts. Scarif, however, reaches EOP up to 8,000% on *S. afghaniensis* (Table 2). In contrast to these broad host-range phages, phage Geonosis is only able to infect two different *Streptomyces* species, its isolation host *S. griseus* and one additional host, *S. olivaceus,* with an EOP of 0.15%, which classifies Geonosis as a narrow host-range phage in this context (Table 2; Fig. 3). Out of the 45 tested *Streptomyces* species, 15 have previously been phylogenetically analyzed (29) and no pattern of phylogenetic relatedness and host range could be observed. Furthermore, an analysis of the defense system repertoire of several of the tested strains was performed using PADLOC (Table S4), and also here, no distinct pattern of defense systems could be detected which would render the host more resistant to phage infection by the here described broad host-range phages (29).

**TABLE 2:** Host range of the *Streptomyces* phages Kamino, Geonosis, Abafar, and Scarif with “efficiency of plating” given in %^*a*^

	Kamino	Geonosis	Abafar	Scarif
*Streptomyces afghaniensis*	**5,000**	0	**16.7**	**8,000**
*Streptomyces albidoflavus* (host)	**6.4**	0	100	100
*Streptomyces antibioticus*	0	0	**2**	0
*Streptomyces celluloflavus*	0	0	0	**2**
*Streptomyces chartreusis*	0	0	0	**2**
*Streptomyces fradiae*	**139.4**	0	0	0
*Streptomyces griseorubens*	0	0	**33.3**	**2,000**
*Streptomyces griseus* (host)	**0.07**	100	0	0
*Streptomyces hygroscopicus*	**0.15**	0	0	0
*Streptomyces kanamyceticus*	**0.04**	0	0	0
*Streptomyces kasugaensis* (host)	100	0	0	0
*Streptomyces longispororuber*	**12.5**	0	0	0
*Streptomyces luridus*	**2,500**	0	**16.7**	**800**
*Streptomyces niveus*	**37.5**	0	**16.7**	0
*Streptomyces nodosus*	0	0	0	**0.2**
*Streptomyces olivaceus*	**0.006**	**0.15**	0	0
*Streptomyces purpurascens*	**2,500**	0	**33.3**	0
*Streptomyces rimosus*	**0.05**	0	0	0
*Streptomyces venezuelae*	**0.02**	0	0	0
*Streptomyces viridosporus*	**1,250**	0	0	0

^*a*^ Underlined numbers indicate the isolation host, and bold numbers indicate productive infection.

### Comparison of genomes

Phage DNA of all four phages was isolated and sequenced using Illumina short read sequencing. Reads were assembled and contigs were annotated using Prokka with implemented PHROG analysis (Table 3). Prediction of the phage lifestyle was performed with the machine-learning tool PhageAI (30) with 93.8% confidence for a temperate lifestyle of Kamino and 93.5%, 95.0%, and 73.6% confidence for Geonosis, Abafar, and Scarif having a virulent lifestyle, respectively.

**TABLE 3:** Summary of genomic features of the four *Streptomyces* phages

Phage name	Host	Genome size (bp)	GC content (%)	Number of ORFs	CDS coding density (%)	Lifestyle prediction	Taxonomy
Kamino	*Streptomyces kasugaensis* DSM 40819	49,381	65.4	73	92.5	Temperate	*Caudoviricetes* *Arquatrovirinae* *Camvirus,* “Camvirus kamino”
Geonosis	*Streptomyces griseus* DSM 40236	57,039	68.9	69	90.2	Virulent	*Caudoviricetes,* “Woodruffvirus geonosis”
Abafar	*Streptomyces albidoflavus* DSM 112524	43,704	60.2	58	85.8	Virulent	*Caudoviricetes*, *Beephvirinae*, *Manuelvirus*, “Manuelvirus abafar”
Scarif	*Streptomyces albidoflavus* DSM 112524	55,306	59.1	83	93.3	Virulent	*Caudoviricetes*, “Scarifvirus,” “Scarifvirus scarif”

**Fig 4:**
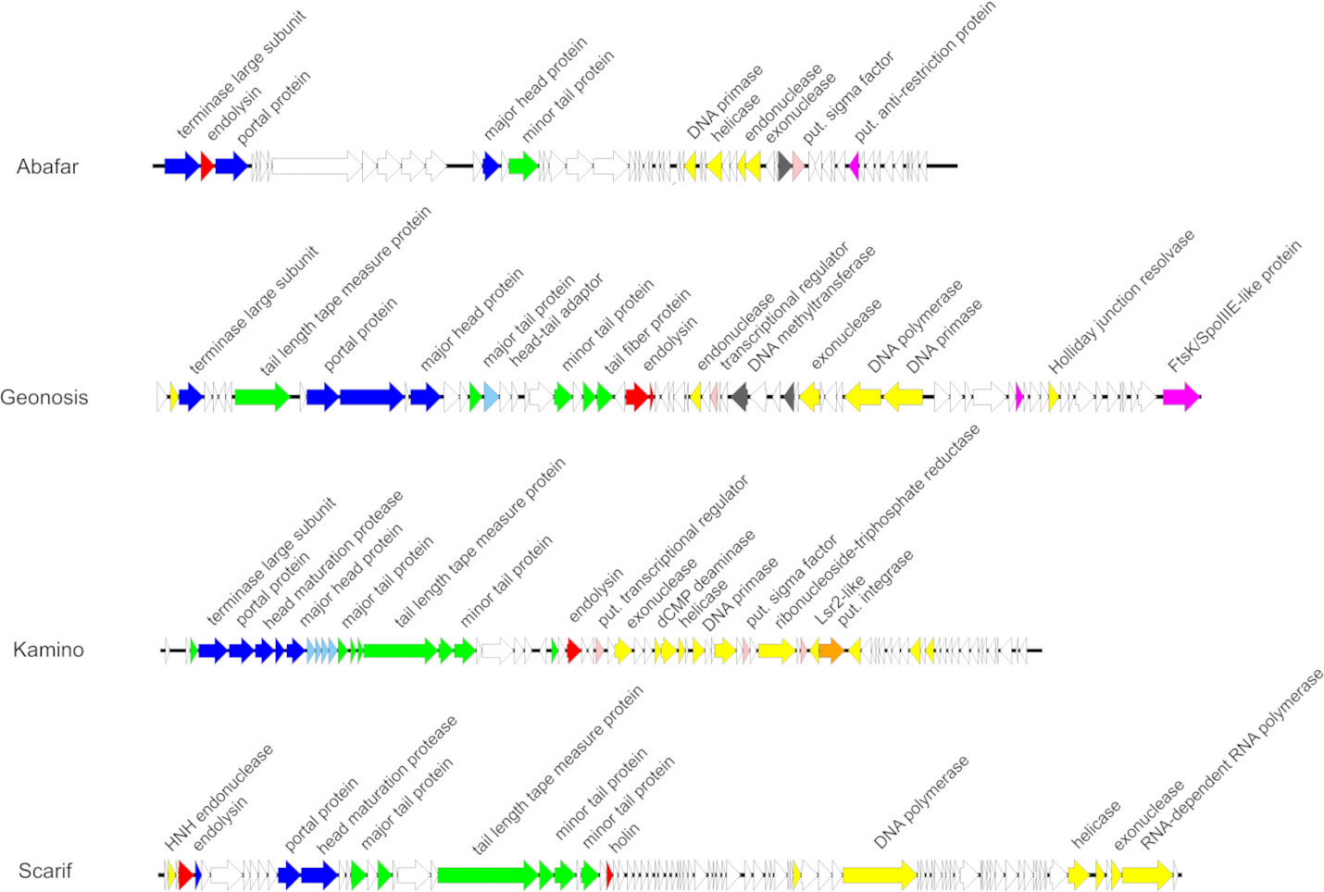
Annotated genomes of phages Abafar, Geonosis, Kamino, and Scarif. Phage Abafar encodes 58 ORFs, Geonosis 69 ORFs, Kamino 91 ORFs, and Scarif 83 ORFs, which are indicated by differently colored arrows. Coloring is based on the PHROG color code for functional clusters (28) (orange, integration and excision; blue, head and packaging; purple, transcription; light blue, connector; green, tail; red, lysis; yellow, DNA, RNA, and nucleotide metabolism; pink, moron, auxiliary metabolic gene, and host takeover; dark gray, other).

The genome of phage Abafar consists of 43,704 bp (GC% 60.2) with 58 predicted open reading frames (ORFs) and 15 genes for tRNAs (Fig. 4), 27 of the 58 ORFs are annotated genes (46%). BLASTn analysis against NCBI database for viruses (taxid: 10239) identified four closely related phages, classified members of the genus *Manuelvirus*. All of them share a similar genome organization with functional gene clusters for packaging and structural proteins with an embedded gene for a putative endolysin between the genes encoding the terminase large subunit and a portal protein (Fig. S2). The cluster for replication contains conserved genes coding for a primase, a helicase, and different nucleases. No genes related to lysogeny were identified, which is in line with the prediction of Abafar being virulent.

Phage Geonosis has a genome size of 57,039 bp with an average GC content of 68.3% comprising 69 ORFs but no tRNA genes (Fig. 4), 24 of the ORFs are annotated genes (34.7%). Comparison with other related Woodruffviruses like phage YDN12 (31) revealed a similar genome organization with gene clusters for packaging, structural head and tail proteins, lysis comprising endolysin and holin genes, and replication containing characteristic genes for an endonuclease, a DNA methyltransferase, an exonuclease, a DNA polymerase, and a DNA primase (Fig. S3). Furthermore, phage Geonosis also harbors genes for a putative Holliday junction resolvase and an FtsK-like protein, respectively, that might also be used during replication.

Phage Kamino features a genome size of 49,381 bp with an average GC content of 65.4% harboring 73 ORFs, of which 33 ORFs are annotated genes (45.2%), but no tRNA genes were found (Fig. 4). BLASTn analysis identified related phages of the genus *Camvirus* like Endor1 (18), phiCAM (32), Verabelle, or Vanseggelen (33). All of them share the same genome organization with characteristic gene clusters for structure, replication, and lysis (Fig. S4). In contrast to the other three isolated phages, phage Kamino harbors a gene for a serine integrase (Kamino_00049) and additional genes putatively involved in transcriptional regulation (Kamino_00029, Kamino_00043 and Kamino_00046) which is in line with the prediction of a temperate lifestyle.

Genomic analysis of phage Scarif revealed a genome size of 55,306 bp (GC%: 59.1) with 89 predicted ORFs, of which 18 are annotated (20.2%), organized in characteristic clusters for replication including genes for a DNA polymerase, a helicase, an exonuclease, or an RNA-dependent RNA polymerase and for head and tail structure comprising genes for a portal protein, minor and major tail proteins, and a tail length tape measure protein (Fig. 4). Given that this protein determines the tail length of the phage and assuming a tail length of 1.5 Å per amino acid residue, the calculated length (~274 nm) aligns with the size range measured via electron microscopy (34). Generally, phage Scarif shares this genomic organization with phages belonging to the genus *Rimavirus* (Fig. S5).

VIRIDIC (Virus Intergenomic Distance Calculator) analysis (Table S3) (35) identified phages Abafar, Geonosis, and Kamino as putative new species within the genera *Manuelvirus*, *Woodruffvirus,* and *Camvirus*, respectively, according to the ICTV rules with 95% and 70% nucleotide sequence identity over the length of the genome as species and genus demarcation criteria, respectively (36, 37). In contrast, based on this analysis, phage Scarif forms a new genus that we suggest to call “Scarifvirus.”

## DISCUSSION

In this study, we present the isolation and characterization of four novel *Streptomyces* phages. Phage Kamino was isolated on *S. kasugaensis,* Geonosis was isolated on *S. griseus,* and Abafar and Scarif were isolated on *S. albidoflavus.* Phage Kamino is predicted to be a temperate phage, carrying a serine integrase on its genome, whereas Geonosis, Abafar, and Scarif are predicted to be strictly virulent. Three of the four phages described in this study have a broad host range with productive infections on 7 to 15 different *Streptomyces* species (out of 45 different strains tested). Out of the 45 strains tested for host range, 15 were previously analyzed for their phylogeny (29); however, no phylogenetic pattern can be observed within the infection pattern. Similar to this, the defense mechanism distribution of the tested strains (Table S4) analyzed by PADLOC does not show a significant pattern (38). In particular, for the strains infected by phage Kamino, we could find only one defense system that was present in all strains, the system PD-T4-6 (39). These strains, however, are all lacking several systems, such as the Ssp (40, 41) or DnD (42) defense systems as well as the cas-type I-B system (43), which are, among others, present in several strains which are resistant to Kamino. We hypothesize that the host-range pattern is therefore rather influenced by receptor availability and cell surface structures than by phylogenetic similarities.

Of the phages reported in this study, Kamino and Geonosis amplify well in liquid cultures (Fig. 2). In particular, Kamino showed highly efficient infection of *S. kasugaensis* already at very low starting titers (10^2^ PFU/mL). In contrast to this, Abafar and Scarif show little amplification in liquid infections and no effect on the growth of their host organism *S. albidoflavus* M145. This is, in fact, not an unusual feature of phages infecting *Streptomyces* and has been reported previously (18, 33). This phenotype could be a phage-specific feature, as it was also described by Hardy et al. (18) in the characterization of five novel *Streptomyces* phages, where, for example, phage Dagobah infecting *S. albidoflavus* M145 does not show a culture collapse despite showing amplification in phage titer, similar to phage Coruscant infecting *Streptomyces venezuelae*, described in the same publication.

When compared to the recently reported *Streptomyces* phages Vanseggelen and Verabelle, which also infect several different species (33) (Fig. S4), two major differences were detected in the genome of phage Kamino (44). First, the gene for the integrase (Kamino_00049) shows no similarity at the nucleotide level to the integrase genes in Vanseggelen and Verabelle; secondly, the gene annotated as a putative tail fiber in Verabelle and Vanseggelen, respectively, also reveals only weak similarities to its homolog in Kamino (Kamino_00020). However, the differences in their tail fibers compared to their otherwise conserved genome structure might explain their different host-range behavior as those structures are major players in virus-host interactions (44). Moreover, broad host-range phages like Kamino, equipped with a serine integrase for a temperate lifestyle, can serve as powerful tools for genetic manipulation across various strains (8, 22). In this context, novel integrative plasmids could be engineered utilizing the attP site of Kamino, expanding the repertoire of genetically modifiable *Streptomyces* strains.

Little is known so far about the characteristic phage traits of different morphotypes in phages infecting actinobacteria (45, 46). In line with our findings, the majority of described *Streptomyces* phages belong to the morphotype of siphoviruses. Phage Abafar isolated in this study, however, might be a good example to study the differences in infection dynamics and adsorption of podoviruses. Genomic analysis of phage Abafar revealed that—in contrast to the three siphoviruses of this study—its genome contains 15 tRNA genes. Those phage-encoded tRNAs are considered to be used to evade host defense mechanisms that directly target tRNAs (47, 48). Furthermore, we detected a gene (Abafar_00064) with an incomplete conserved domain that shows similarities to an ArdA-like anti-restriction protein. BLASTp analysis of the predicted amino acid sequence identified homologous proteins only in other podoviruses infecting *Streptomyces* species.

To gain deeper insights into the determinants of phage-host interactions, particularly in relation to sensitivity to antiphage defense systems—including those involving small molecules—the comparative analysis of a diverse set of phages is required. In fact, systematic phage collections proved highly valuable in assessing the efficacy of diverse defense systems against a wide spectrum of phages infecting a particular host or genus (49). Broad host-range phages further provide the opportunity to determine context dependency by comparing the effect of a given defense system/antiphage molecule across different host species. Beyond the study of bacterial immune systems and phage-encoded counter-defenses, the application of phages has also provided new insights into cell envelope biosynthesis and structure through the screening and analysis of phage-resistant bacterial strains (14, 50, 51). Expanding the phage universe by providing new isolates to the scientific community is therefore valuable across multiple research areas within phage-host biology.

## SUPPLEMENTAL MATERIAL

Supplemental tables and figures spectrum.00663-24-s0001.pdf Tables S1 and S2; Fig. S1 to S5.

Table S3 spectrum.00663-24-s0002.xlsx VIRIDIC analysis of phages.

Table S4 spectrum.00663-24-s0003.xlsx PADLOC analysis of phage defense systems.

ASM does not own the copyrights to Supplemental Material that may be linked to, or accessed through, an article. The authors have granted ASM a non-exclusive, world-wide license to publish the Supplemental Material files. Please contact the corresponding author directly for reuse.
