# Correction for Rackow et al., “Isolation and characterization of the new *Streptomyces* phages Kamino, Geonosis, Abafar, and Scarif infecting a broad range of host species”

**DOI:** 10.1128/spectrum.00297-25

**Published:** 2025-06-30

**Authors:** Bente Rackow, Clara Rolland, Isabelle Mohnen, Johannes Wittmann, Mathias Müsken, Jörg Overmann, Julia Frunzke

## AUTHOR CORRECTION

Volume 12, no. 11, e00663-24, 2024, https://doi.org/10.1128/spectrum.00663-24. The species *Streptomyces albidoflavus* was incorrectly annotated and should read "*Streptomyces coelicolor* A3(2)" throughout. The genome sequences and phage isolate characterizations remain valid, and the overall results and conclusions of the study are unaffected by this taxonomic correction.

