# Supplementary material for: Isolation and characterization of the new Streptomyces phages Kamino, Geonosis, Abafar, and Scarif infecting a broad range of host species
Source: Microbiol Spectr. 2024 Sep 25;12(11):e00663-24. doi: 10.1128/spectrum.00663-24 (PMC11536984; doi:10.1128/spectrum.00663-24)
Supplement: Supplemental tables and figures — Tables S1 and S2; Fig. S1 to S5. [file spectrum.00663-24-s0001.pdf]

Supplementary Information to:

Isolation and characterization of the new *Streptomyces* phages Kamino, Geonosis, Abafar and Scarif infecting a broad range of host species

Bente Rackow[1], Clara Rolland[2], Isabelle Mohnen[1], Johannes Wittmann[2], Mathias Müsken[3], Jörg Overmann[2], and Julia Frunzke [1]*

[1]Institute of Bio- and Geosciences, Forschungszentrum Jülich, Germany

[2]Leibniz Institute DSMZ—German Collection of Microorganisms and Cell Cultures, Braunschweig, Germany

[3]Central Facility for Microscopy, Helmholtz Centre for Infection Research, Braunschweig, Germany

*Corresponding author:

Julia Frunzke; Email: j.frunzke@fz-juelich.de; Phone: +49 2461 615430

Content:

Table S1: Bacterial strains used for host range analysis and efficiency of plating

Table S2: Bacterial strains used for host range analysis.

Supplementary Figure 1

Supplementary Figure 2

Supplementary Figure 3

Supplementary Figure 4

Supplementary Figure 5

Table S3: VIRDIC analysis of phages (extra)

VIRDIC analysis of phage genuses with comparison of nucleotide sequence identity to other known *Streptomyces* phages. Colouration indicates sequence identity of phages, with red showing no identity, orange and yellow indicating low percentage identity and green indicating high percentage

of identity. The newly isolated and characterized phages were clustered into species and genera according to ICTV rules with 95 % and 70 % nucleotide sequence identity over the length of the genome respectively. The numbers in the table show the percentage of nucleotide sequence identity.

Table S4: PADLOC analysis of host defense systems (extra)

PADLOC analysis of 34 of the 45 tested *Streptomyces* species for their composition of defense systems. 0 indicates that the respective defense system is absent in the species and 1 indicates that the defense system was found encoded in the genome of the respective species. Abbreviations of defense systems are according to the PADLOC tool.

Supplementary Information

Table S1: Bacterial strains used for host range analysis and efficiency of plating.

Bacterial strains in this table were used to perform detailed experiments in the sections describing phage isolation and propagation, infection dynamics, plaque development and phage host range assay with EOP.

**Strains**	**Genotype**	**Reference**
Streptomyces afghaniensis	WT	DSM 40228
Streptomyces albaduncus	WT	DSM 40478
Streptomyces albidoflavus	WT (*S. coelicolor* is a heterotypic synonym of this strain)	DSM 112524
Streptomyces albidoflavus M145 (host Abafar & Scarif)	*S. coelicolor* A3(2) lacking plasmids SCP1 and SCP2	(Bentley *et al.*, 2002)
Streptomyces albulus	WT	DSM 40492
Streptomyces antibioticus	WT	DSM 40234
Streptomyces avermitilis	WT	DSM 46492
Streptomyces azureus	WT	DSM 40106
Streptomyces celluloflavus	WT	DSM 40839
Streptomyces chartreusis	WT	DSM 40085
Streptomyces clavuligerus	WT	DSM 738
Streptomyces fimbriatus	WT	DSM 40942
Streptomyces fradiae	WT	DSM 40063
Streptomyces galbus	WT	DSM 40089
Streptomyces griseofuscus	WT	DSM 40191
Streptomyces griseorubens	WT	DSM 40160
Streptomyces griseus (host Geonosis)	WT	DSM 40236
Streptomyces humidus	WT	DSM 40263
Streptomyces hygroscopicus	WT	DSM 40578
Streptomyces inusitatus	WT	DSM 41441
Streptomyces kanamyceticus	WT	DSM 40500
Streptomyces kasugaensis (host Kamino)	WT	DSM 40819
Streptomyces lavendulae	WT	DSM 40069
Streptomyces longispororuber	WT	DSM 40599

Streptomyces luridus	WT	DSM 40081
Streptomyces niveus	WT	DSM 40088
Streptomyces nodosus	WT	DSM 40109
Streptomyces olivaceus	WT	DSM 41536
Streptomyces purpurascens	WT	DSM 40310
Streptomyces rimosus	WT	DSM 40260
Streptomyces sulfonofaciens	WT	DSM 41679
Streptomyces thioluteus	WT	DSM 40027
Streptomyces venezuelae NRRL B-65442	WT	DSM 112328
Streptomyces violaceus	WT	DSM 40082
Streptomyces *viridosporus*	WT	DSM 40243

Table S2: Bacterial strains used for host range analysis.

Table S2 includes all *Streptomyces* strains which were used for the host range assay. Strains which showed productive infection were further tested and are mentioned in Table S1 in detail

***Streptomyces* strains used for the host range**	**DSM Number**
Streptomyces afghaniensis	DSM 40228
Streptomyces albaduncus	DSM 40478
Streptomyces albidoflavus	DSM 112524
Streptomyces alboniger	DSM 40043
Streptomyces albulus	DSM 40492
Streptomyces anandii	DSM 40535
Streptomyces antibioticus	DSM 40234
Streptomyces avermitilis	DSM 46492
Streptomyces avidinii	DSM 40526
Streptomyces azureus	DSM 40106
Streptomyces bluensis	DSM 40564

Streptomyces celluloflavus	DSM 40839
Streptomyces chartreusis	DSM 40085
Streptomyces chrestomyceticus	DSM 40545
Streptomyces clavuligerus	DSM 738
Streptomyces echinatus	DSM 40013
Streptomyces eurocidicus	DSM 40604
Streptomyces fradiae	DSM 40063
Streptomyces fimbriatus	DSM 40942
Streptomyces galbus	DSM 40089
Streptomyces griseus	DSM 40236
Streptomyces griseofuscus	DSM 40191
Streptomyces griseorubens	DSM 40160
Streptomyces humidus	DSM 40263
Streptomyces inusitatus	DSM 41441
Streptomyces kanamyceticus	DSM 40500
Streptomyces kasugaensis	DSM 40819
Streptomyces lavendulae	DSM 40069
Streptomyces litmocidini	DSM 40164
Streptomyces longispororuber	DSM 40599
Streptomyces luridus	DSM 40081
Streptomyces mutomycini	DSM 41691
Streptomyces niveus	DSM 40088
Streptomyces nodosus	DSM 40109
Streptomyces olivaceus	DSM 41536

Streptomyces purpurascens	DSM 40310
Streptomyces rapamycinicus	DSM 41530
Streptomyces rimosus	DSM 40260
Streptomyces scabiei	DSM 41658
Streptomyces sulfonofaciens	DSM 41679
Streptomyces thioluteus	DSM 40027
Streptomyces venezuelae	DSM 112328
Streptomyces violaceus	DSM 40082
Streptomyces viridosporus	DSM 40243
Streptomyces wellingtoniae	DSM 40632

Supplementary Figure 1

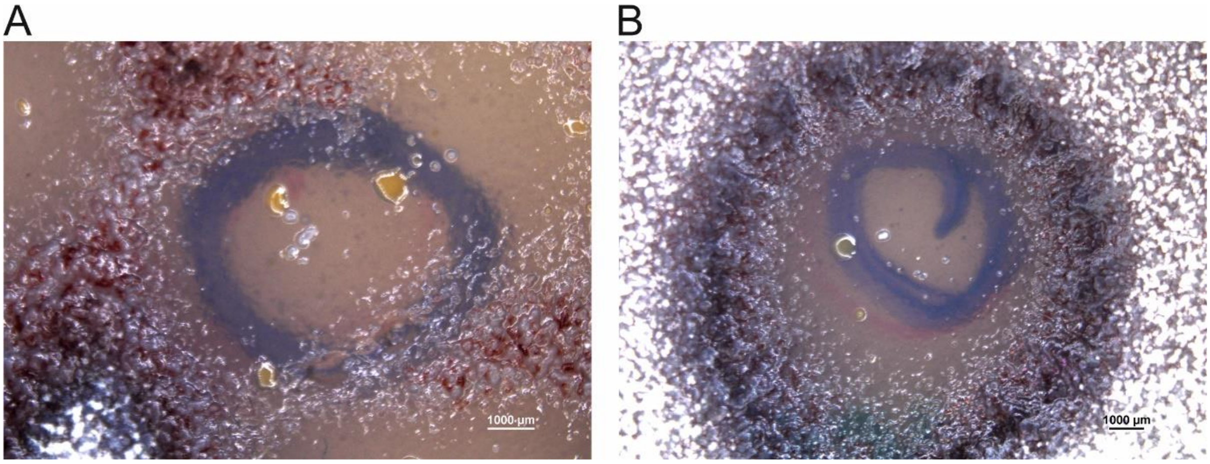

Figure S1: Plate images of phage Abafar (A) and phage Scarif (B) 72 hours post infection; production of secondary metabolites (e.g. actinorhodin as blue pigmentation) at the plaque interface. The blue drawing from underneath the plate marks the initial plaque location. White coloration of the surrounding bacterial lawn indicates sporulation. The scale bar is 1000 µm.

50 Supplementary Figure 2

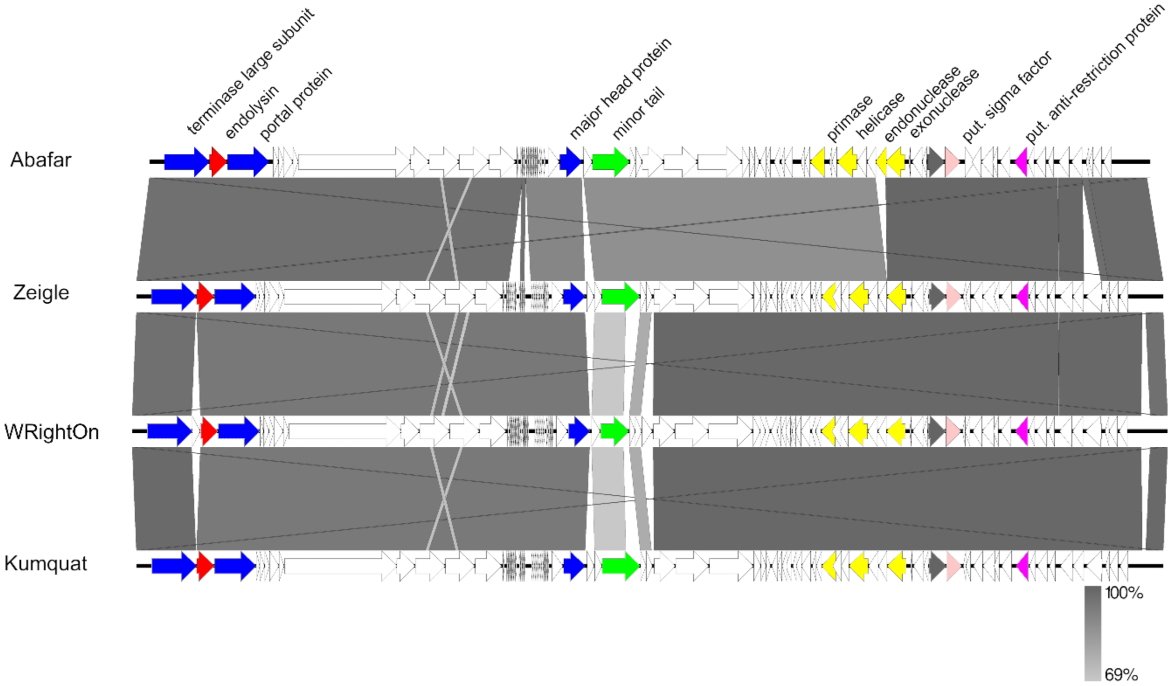

Figure S2: Synteny plot of phage Abafar compared to other manuelviruses at the nucleotide level. The figure was generated with EasyFig (Sullivan et al., 2011). Colouring is based on the PHROG colour code for functional clusters (Terzian et al., 2021) (orange: integration and excision; blue: head and packaging; purple: transcription; light blue: connector; green: tail; red: lysis; yellow: DNA, RNA and nucleotide metabolism; pink: moron, auxiliary metabolic gene and host takeover; dark grey: other).

51 Supplementary Figure 3

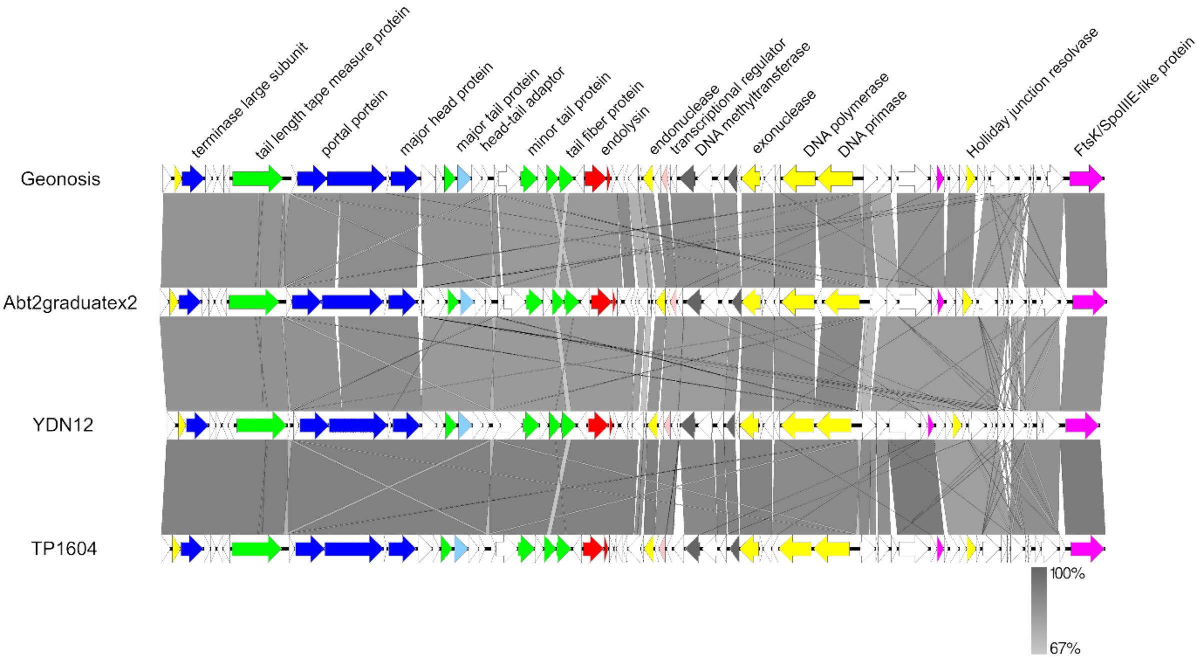

Figure S3: Synteny plot of phage Geonosis compared to other woodruffviruses at the nucleotide level. The figure was generated with EasyFig (Sullivan et al., 2011). Colouring is based on the PHROG colour code for functional clusters (Terzian et al., 2021) (orange: integration and excision; blue: head and packaging; purple: transcription; light blue: connector; green: tail; red: lysis; yellow: DNA, RNA and nucleotide metabolism; pink: moron, auxiliary metabolic gene and host takeover; dark grey: other).

Supplementary Figure 4

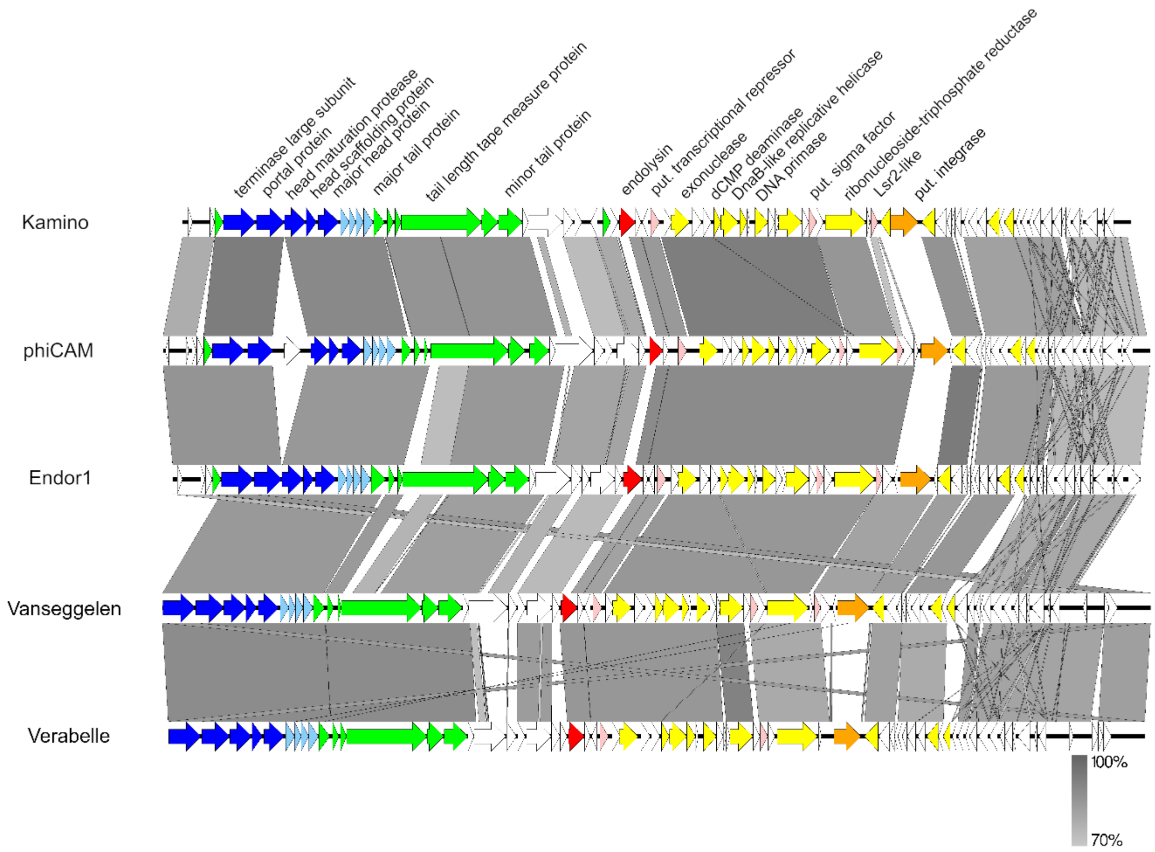

Figure S4: Synteny plot of phage Kamino compared to other camviruses at the nucleotide level. The figure was generated with EasyFig (Sullivan et al., 2011). Colouring is based on the PHROG colour code for functional clusters (Terzian et al., 2021) (orange: integration and excision; blue: head and packaging; purple: transcription; light blue: connector; green: tail; red: lysis; yellow: DNA, RNA and nucleotide metabolism; pink: moron, auxiliary metabolic gene and host takeover; dark grey: other).

Supplementary Figure 5

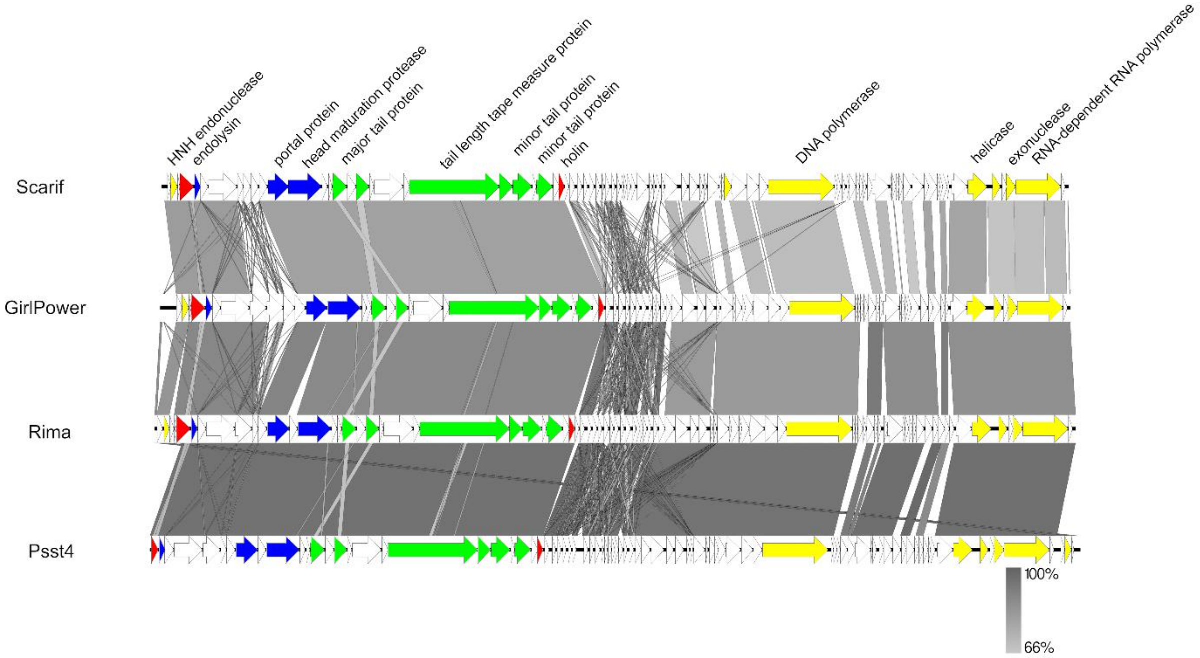

Figure S5: Synteny plot of phage Scarif compared to closely related members of Rimavirus at the nucleotide level. The figure was generated with EasyFig (Sullivan et al., 2011). Colouring is based on the PHROG colour code for functional clusters (Terzian et al., 2021) (orange: integration and excision; blue: head and packaging; purple: transcription; light blue: connector; green: tail; red: lysis; yellow: DNA, RNA and nucleotide metabolism; pink: moron, auxiliary metabolic gene and host takeover; dark grey: other).
